# *In Vitro* Ultramorphological Assessment of Apoptosis on CEMss Induced by Linoleic Acid-Rich Fraction from *Typhonium flagelliforme* Tuber

**DOI:** 10.1093/ecam/neq010

**Published:** 2011-05-02

**Authors:** Syam Mohan, Ahmad Bustamam, Siddig Ibrahim, Adel S. Al-Zubairi, Mohammed Aspollah, Rasedee Abdullah, Manal Mohamed Elhassan

**Affiliations:** ^1^UPM-MAKNA Cancer Research Laboratory, Institute of Bioscience, 43400 UPM Serdang, University Putra Malaysia, Malaysia; ^2^Department of Clinical Biochemistry, University of Sana'a, Sana'a, Yemen; ^3^Department of Chemistry, Faculty of Science, 43400 UPM Serdang, University Putra Malaysia, Malaysia; ^4^Faculty of Veterinary Medicine, 43400 UPM Serdang, University Putra Malaysia, Malaysia

## Abstract

The plant *Typhonium flagelliforme*, commonly known as “rodent tuber” in Malaysia, is often used as a health supplement and traditional remedy for alternative cancer therapies, including leukemia. This study aimed to evaluate *in vitro* anti-leukemic activity of dichloromethane extract/fraction number 7 (DCM/F7) from *T. flagelliforme* tuber on human T4 lymphoblastoid (CEMss) cell line. The DCM extract of tuber has been fractionated by column chromatography. The obtained fractions were evaluated for its cytotoxicity toward CEMss cells as well as human primary blood lymphocytes (PBLs). Assessment of apoptosis produced by the most active fraction was evaluated by various microscopic techniques and further confirmation of apoptosis was done by terminal deoxynucleotidyl transferase dUTP nick end labeling (TUNEL) assay. Phytochemical screening was done by gas chromatography-mass spectrometry (GC-MS). The results shows that 7 out of 12 fractions showed significant cytotoxicity against the selected cell line CEMss, in which fractions DCM/F7, DCM/F11 and DCM/F12 showed exceptional activity with 3, 5 and 6.2 μg ml^−1^, respectively. Further studies in the non-cancerous PBL exhibited significant selectivity of DCM/F7 compared to other fractions. Cytological observations showed chromatin condensation, cell shrinkage, abnormalities of cristae, membrane blebbing, cytoplasmic extrusions and formation of apoptotic bodies as confirmed collectively by double-staining of acridine orange (AO)/propidium iodide (PI), SEM and TEM. In addition, DCM/F7 has increased the cellular DNA breaks on treated cells. GC-MS revealed that DCM/F7 contains linoleic acid, hexadecanoic acid and 9-hexadecanoic acid. The present results indicate that *T. flagelliforme* possess a valuable anti-leukemic effect and was able to produce distinctive morphological features of cell death that corresponds to apoptosis.

## 1. Introduction

The genus *Typhonium* gained favorable taxonomic and pharmacological attention recently. This plant is a member of the aroid family. The distinguishing feature of aroids is the occasionally beautiful and often bizarre combination of spathe and spadix called the inflorescenceis, which is used for trapping their pollinators because of their particular morphology and organization of their inflorescences [1]. *Typhonium* species are common in Malaysian lowlands, frequently found in disturbed places [2]. The plant *T. flagelliforme*, commonly known as “rodent tuber” in Malaysia, is often used as a traditional remedy for alternative cancer therapies, including leukemia, by various ethnic populations [3]. This plant is widely distributed in soft, damp and shady habitats in Southeast Asia, extending even to Northern Australia and South India [4]. *Typhonium flagelliforme* is now commercially available as a healthcare supplement to cure breast, lung, rectum, liver, prostate, pancreas and cervical cancers and leukemias [5].

Recently a bioassay-guided approach demonstrated that several fractions of hexane and DCM extracts of *T. flagelliforme* were found to inhibit the growth of NCI-H23 non-small cell lung carcinoma cell line significantly; however, most of these active fractions were also found to inhibit the growth of non-tumorigenic Bagg Albino (BALB)/c 3T3 mouse fibroblast cell line [4]. A previous study had reported that the hexane extract of *T. flagelliforme* displayed poor cytotoxic activity against *in vitro* P388 murine leukemia cells [6]. A low cytotoxic activity has been exhibited by the polar fraction of this plant *in vitro* [7] and found to provide relief in cough and asthma [8]. Pharmacological studies conducted on rats also indicated that *T. flagelliforme* juice extract could prevent hepatocarcinogenesis [4].

Plants as natural products are valuable sources of bioactive compounds and have been used in almost all cultures and communities for thousands of years [9]. Previous phytochemical studies with the *T. flagelliforme* revealed several chemical constituents. The hexane extract was reported to contain saturated hydrocarbons and aliphatic acids [7], while the ethyl acetate extract was found to contain aromatic fatty acids [10], the DCM extract of whole plants exhibits the presence of hexadecanoic acid, 1-hexadecene, phytol and a derivative of phytol [4]. In addition, phenylpropanoid glycosides, sterols and a cerebroside which has anti-hepatotoxic activity were also reported from the root of this plant [11].

Alternative medicines are used widely all over the world [12, 13]. Some natural plants are currently being used extensively as food and health supplements, which help to combat cancer/tumor and stimulate immunity. In any case, the use of complementary and alternative medicines has not been as popular in modern medicine until recently. This may be most probably due to the lack of enough biological evidence stating their functional mechanisms. But for the past few decades, the value of complementary and alternative medicine (CAM) has been rediscovered by many modern scientific researchers, and numerous studies have established the potential use of herbal preparations [14, 15]. However, much study is needed to understand further the use of alternative therapy, including healthcare food supplements (vitamins, antioxidants and fatty acids) and herbal preparation in treating cancer, particularly the benefits and risks in using such edible plant therapy for treatment. There is very little literature available on the mechanism of *T. flagelliforme* and its effects on other cancers. In this study, we aimed to establish the relation between this plant and its anticancer effect toward leukemia, in regard to its mechanism of cell death and possible phytochemicals present.

## 2. Methods

### 2.1. Plant Materials

*Typhonium flagelliforme* (Lodd.) Blume (Araceae) whole plant (leaves and tubers) were collected in July 2008 from the state of Selangor, Malaysia. Authentication was done at the Faculty of Science, University Putra Malaysia, where the voucher specimen TF-TL100156 was deposited.

### 2.2. Extraction of *T. flagelliforme* Tuber

Fresh plants were harvested and washed thoroughly with running tap water and then distilled water, followed by tubers being separated from aerial parts prior to drying. The tubers were air dried and then oven dried (Memmert, model 100-800, Schwabach, Germany) at lower temperature. The fully dried tubers were powdered and weighed prior to cold maceration. The powdered tubers were then extracted with hexane to remove the non-polar fractions and then treated with DCM. The DCM extraction was done for 7 days with occasional shaking (Edmund Buchler shaker, Model KS-15, Hechingen, Germany) and the process was repeated 3 times. The combined extracts were filtered through Whatman No. 41 filter paper (pore size: 20–25 *μ*m) and vacuum dried using a rotary evaporator (Buchi, R-210, Postfach, Switzerland) and weighed to calculate the yield of the extracts. The extracts were kept at 4°C until further requirement.

### 2.3. Fractionation of the DCM Extract

The DCM extract (2 g) from *T. flagelliforme* tuber was fractionated by vacuum liquid chromatography (VLC). The stationary phase was made up of glass column packed with silica gel 60 PF_254_ with gypsum (Merck, Darmstadt, Germany). The mobile phase consisted of combinations of hexane, ethyl acetate and methanol and the eluting strength of the solvent was increased gradually by increasing the composition of a more polar solvent. For the purification of extract, the initial solvent composition was hexane-ethyl acetate (9 : 1 v/v; 1000 ml), which was then changed to hexane-ethyl acetate (7 : 3 v/v; 1000 ml), followed by hexane-ethyl acetate (1 : 1 v/v; 1000 ml), ethyl acetate (500 ml) and finally methanol (500 ml). The eluent was collected in fractions of 100 ml. The chemical composition of each fraction was evaluated using thin-layer chromatography (TLC) and visualized with ultraviolet (UV) light (254 nm and 365 nm). Based on the TLC profiles, those fractions with similar compositions were pooled together and concentrated under reduced pressure. A total of 12 combined fractions were obtained and designated as DCM/F1, DCM/F2,…, DCM/F12. The yield value of each fraction was recorded (Table 1). 

### 2.4. Cell Viability Assay

Human T4-lymphoblastoid cell line CEMss was provided from the National Institutes of Health (NIH) AIDS Research and Reference Reagent Program, Division of AIDS, NIAID, NIH: USA, and was used in this study (Order No: 20081911). The cell suspension (0.1 million cells ml^−1^) was plated out into 96-well microtiter plates. All the 12 fractions were dissolved with dimethylsulfoxide (DMSO) and the final concentration of DMSO was 0.1% (v/v). Different concentrations of the sample were prepared with serial dilution. Dimethylsulfoxide (0.1%) was used as a control.

The toxicity profiles of the fraction were assessed using the 3-[4, 5-dimethylthiazol-2-yl]-2, 5-diphenyltetrazolium bromide (MTT) microculture tetrazolium viability assay [16]. Thereafter, the various concentrations of samples were plated out in triplicates. Each plate included untreated cell controls and a blank cell-free control. After 68 h of incubation, MTT (5 mg ml^−1^) was added to each well and the plates incubated for a further 4 h and the media removed. DMSO was later added into each well to solubilize the formazan crystals. The absorbance was read at a wavelength of 595 nm using a microtiter plate reader (Tecan Sunrise basic, Groedig, Austria).The percentage cellular viability was calculated with the appropriate controls taken into account. The concentration which inhibited 50% of cellular growth (IC_50_ value) was determined. All experiments for each extract were carried out in triplicates. The inhibitory rate of cell proliferation was calculated by the following formula: growth inhibition = OD control − OD treated/OD control × 100. The cytotoxicity of the sample on cancer cells was expressed as IC_50_ values (the drug concentration reducing the absorbance of treated cells by 50% with respect to untreated cells).

### 2.5. The Effect of Selected Fractions on Stimulated (Proliferated) Primary Human Blood Lymphocytes

Since the crude DCM extract exhibited an IC_50_ value of 6 *μ*g ml^−1^, those fractions of the DCM extract that showed IC_50_ value below this concentration were used in a study toward normal cells, as described previously by Shaun et al. [17] with minor modifications. Hence to compare the effects of the selected fractions, DCM/F7, DCM/F11 and DCM/F12 on the leukemic (CEMss) cells to its effects on equivalent non-cancer cells, their impact on primary human blood lymphocytes (PBLs) was then investigated. Centrifuge tubes (size: 25 ml) were filled with 10 ml lymphocyte separation medium (PAA, Pasching, Austria). Undiluted heparinized whole blood was then carefully poured over the lymphocyte-separation solution. The separation process was performed by centrifugation at 1200 × g for 20 min. The lymphocytes (70–100% enrichment) were concentrated in the interphase (white layer) between the plasma and the separation solution. They were subsequently extracted carefully using a sterile Pasteur pipette and washed twice in culture medium. The culture media used was Quantum PBL media containing 10% Fetal Bovine Serum (FBS) supplemented with 100 U ml^−1^ penicillin, 100 *μ*g ml^−1^ streptomycin and phytohemagglutinin (PHA) 10 mM (PAA, Pasching, Austria) at 37°C in a 5% carbon dioxide (CO_2_) atmosphere. Flasks were seeded with 1 × 10^6^ of cells and treated by fractions with the respective IC_50_ concentration toward the CEMss cells for 72 h. A negative control of 0.1% v/v DMSO was used. Three independent separate experiments were carried out for each exposure. After 72 h of exposure, cells were harvested followed by testing for the evidence of cytotoxicity toward lymphocytes cells which was carried out by Trypan blue exclusion assay. The trypan blue exclusion assay detects only dead cells with damaged cell membrane (necrotic cells), since trypan blue does not penetrate into living cells [18].

### 2.6. Identification and Chemical Analysis of Bioactive Fraction Using GC-MS

The bioactive DCM/F7 was analyzed by Shimadzu GC-MS GC-17A. A BP-X5 capillary column (30 m 0.25 mm I.D. 0.25 lm film thickness) was used for gas chromatographic separation of the analytes. The injection volume was 1 *μ*l with a split ratio of 25 : 1; the injector temperature was held constant at 250°C. Helium was used as the carrier gas with an inlet pressure of 46.9 kPa, corresponding to a flow rate of 1.0 ml min^−1^. The column oven temperature was set at 35°C (held for 3 min), raised at 8°C min^−1^ to 280°C (held for 3 min) and finally held at 300°C for 15 min. The mass spectrometer was operated in the electron impact (EI) mode with ionization energy of 70 eV. The transfer line was set at 290°C. The chemical constituents of the analyte were identified by comparing the MS fragmentation patterns with those of NIST/EPA/NIH mass special database library of the GC-MS system.

### 2.7. Microscopic Observation of Cellular Morphology Using Phase-Contrast Inverted Microscope

This analysis examines whether apoptosis may be implicated in mediating cell death among CEMss cells elicited by fractions of *T. flagelliforme* tuber. The DCM/F7 was chosen in this analysis, since this fraction showed the lowest IC_50_ with higher selectivity compared with other fractions. Morphological appearance of treated cells was compared with the untreated control by using the normal inverted microscope. The morphological changes of the cells were observed under the normal inverted microscope post treatment [19]. The IC_50_ value of the DCM/F7 was subsequently used in this study. Human T4-lymphoblastoid cell line (CEMss) were treated with the fraction for 48 and 72 h. Untreated cells served as the negative control.

### 2.8. Quantification of Apoptosis Using Propidium Iodide and Acridine Orange Double-Staining

DCM/F7-induced cell death in CEMss leukemia cells was quantified using propidium iodide (PI) and acridine orange (AO) double-staining according to standard procedures and examined under fluorescence microscope (Lieca attached with Q-Floro Software). Briefly, treatment was carried out in a 25 ml culture flask (Nunc, Roskilde, Denmark). CEMss cells were plated at concentration of 1 × 10^6^ cell ml^−1^ and treated with DCM/F7 at IC_50_ concentration. Flasks were incubated in atmosphere of 5% CO_2_ at 37°C for 24 and 48 h. The cells were then spin down at 300 × g for 10 min. Supernatant was discarded and the cells were washed twice using phosphate buffered saline (PBS) after centrifuging at 300 × g for 10 min to remove the remaining media. Ten microliters of fluorescent dyes containing AO (10 *μ*g ml^−1^) and PI (10 *μ*g ml^−1^) were added into the cellular pellet at equal volumes. Freshly stained cell suspension was dropped into a glass slide and covered by a cover slip. Slides were observed under UV-fluorescence microscope within 30 min before the fluorescent color starts to fade. The percentages of viable, early apoptotic, late apoptosis and secondary necrotic cells were determined in >200 cells. AO and PI are intercalating nucleic acid-specific fluorochromes which emit green and orange fluorescence, respectively, when they are bound to DNA. Of the two, only AO can cross the plasma membrane of viable and early apoptotic cells. The criteria for identification are as follows: (i) viable cells appear to have green nucleus with intact structure; (ii) early apoptosis exhibit a bright-green nucleus showing condensation of chromatin in the nucleus; (iii) dense orange areas of chromatin condensation showing late apoptosis and (iv) orange intact nucleus depicting secondary necrosis [20]. This assay provides a useful quantitative evaluation and was done in triplicates (*n* = 3).

### 2.9. Exterior Ultrastructural Effects of DCM/F7 on CEMss Cells (SEM)

CEMss cells were cultured with the IC_50_ of DCM/F7 and incubated for 24 and 48 h. The cancer cells were centrifuged for 10 min at 500 × g. The pellets were fixed in 4% (v/v) glutaraldehyde in 0.1 M coccadylate buffer (pH: 7.4) for 4 h, 4°C. The fixed cells were washed in three changes of sodium coccadylate buffer for 10 min each, post-fixed in 1% osmium tetraoxide at 4°C. The specimens were then washed in three changes of sodium coccadylate buffer (pH: 7.4) for 10 min each, dehydrated in ascending grades of acetone (35, 50, 75, 95 and 100%), and brought to critical point of drying by the critical point drier (CPD 030, Bal-TEC, Balzer, Switzerland) for 30 min. The cells were affixed to a metal SEM stub and sputter-coated in gold by using SEM coating unit (E5100 Polaron, west Sussex, UK). The coated specimens were viewed using scanning electron microscope (JOEL 64000, Tokyo, Japan) at accelerating voltage of 15–25 kV.

### 2.10. Transmission Ultrastructural Effects of DCM/F7 on CEMss Cells (TEM)

CEMss cells were cultured with the IC_50_ of DCM/F7 and incubated for 24 and 48 h at 37°C. The cultured cells were harvested and centrifuged for 10 min at 500 × g at room temperature. The pellets were fixed in 4% (v/v) glutaraldehyde in 0.1 M coccadylate buffer (pH 7.4) for 4 h at 4°C. The fixed cells were centrifuged, and the pellets were blocked in serum which was later fixed in glutaraldeyde overnight at 4°C. The specimens were washed in three changes of sodium coccadylate buffer (pH 7.4) for 10 min each, post-fixed in 1% osmium tetraoxide at 4°C. The specimens were then washed in three changes of sodium coccadylate buffer (pH: 7.4) for 10 min each and dehydrated with a graded series of acetone (35, 50, 75, 95 and 100%). The cells were then infiltrated with acetone and resin and embedded with 100% resin in beam capsule, and left to polymerize at 60°C for 48 h. The area of interest in the embedded cells resin block was chosen using the toulidine blue staining and later examined using light microscope. The selected area was cut in ultrathin sections using an ultra microtome. The sections were placed into a grid and stained with uranyl acetate for 10 min followed by 50% filtered acetone, and finally stained using lead which was then washed twice with distilled water. The stained samples were then viewed under transmission electron microscope (Phillips, Eindhoven, The Netherlands).

### 2.11. ApoBrdU-Terminal Deoxynucleotidyl Transferase dUTP Nick End Labeling Assay

The induction of apoptosis following incubation of DCM/F7 on CEMss cells was evaluated with the terminal deoxynucleotidyl transferase dUTP nick end labeling (TUNEL) assay according to the manufacturer's protocol (APO-BRDU kit, Sigma, St Louis, Missouri.). Briefly, about 1 × 10^6^ exponentially growing cells were seeded and treated with the DCM/F7 for 12, 24 and 48 h. The cultured cells were harvested and, following two washes in 1 ml of PBS, cells were fixed in 1% paraformaldehyde in PBS and placed on ice for 15 min and then fixed with 5 ml of 70% (v/v) ethanol on ice for 30 min. Following washing in cold-wash buffer (APO-BRDU kit, Sigma), cells were incubated for 60 min at 37°C in a temperature-controlled bath with occasional shaking in the freshly prepared DNA-labeling solution (APO-BRDU kit, Sigma) containing Br-dUTP and TdT enzyme. At the end of the incubation, cells were rinsed twice with rinsing buffer (APO-BRDU kit, Sigma), later incubated for 30 min at room temperature in the dark with 0.1 ml of the antibody solution containing anti-BrdUFITC monoclonal antibody. Following incubation with the antibody, a solution of PI/RNase was added to the cell suspension for 30 min at room temperature in the dark. Cells were then analyzed by a Dako flow cytometer equipped with an Argon laser (Cyan ADP, Dako, Denmark), where the analysis was performed using Summit V4.3 software.

### 2.12. Statistical Analysis

Results were reported as mean ± SD for at least three analyses for each sample. Normality and homogeneity of variance assumptions were checked. Statistical analyses were performed according to the Statistical Package for Social Sciences, Version 16 (SPSS-16.0) package. Analyses of variance were performed using the Analysis of Variance (ANOVA) procedure. The Pearson chi-square test was used to analyze the data of the comparison of selectivity of different fractions on PBL. Significant differences (*P* < .05) between the means were determined using Duncan's multiple-range tests.

## 3. Results

### 3.1. *In Vitro* Cytotoxicity against CEMss and Stimulated Primary Human Blood Lymphocytes

**Figure 1:**
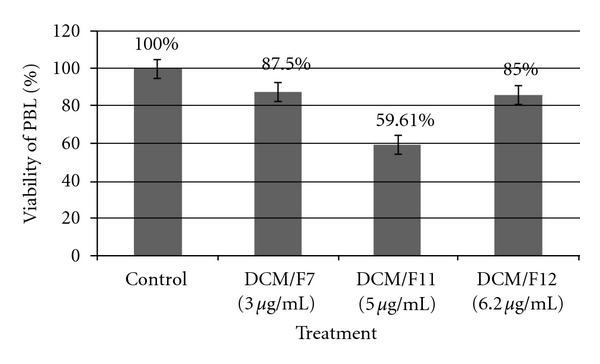
Inhibition effects of selected fraction (DCM/F7, DCM/F11 and DCM/F12) on proliferated human peripheral blood lymphocytes. Results are means ± SD for *n* = 3.

Fractionation of the DCM extract of the plant tuber by vacuum liquid column chromatography yielded a total of 12 fractions. A total of 7 out of 12 fractions showed significant cytotoxicity against the selected cells line, CEMss (Table 1). The MTT results showed that fractions DCM/F7, DCM/F11 and DCM/F12 exhibited exceptional activity with 3, 5 and 6.2 *μ*g ml^−1^, respectively, which was below the cytotoxicity level of crude DCM extract. Comparatively, 5-fluorouracil, a drug with antineoplastic activity was used in this study. 5-fluorouracil is used widely in the treatment of leukemia and it imposed an inhibitory effect on CEMss cells with an IC_50_ value of 1.43 ± 0.06 *μ*g ml^−1^. In order to further evaluate the mechanistic study of the fractions, we compared the cytotoxicty of DCM/F7 against DCM/F11 and DCM/F12 on non-tumorigenic cells (human PBL). Interestingly, in contrast to the effects of 3 *μ*g ml^−1^ DCM/F7 on CEMss, which killed half of the cell population within 72 h, there was no evidence of significant cytotoxicity on human PBL. DCM/F7, furthermore, exhibited very less cytotoxicity on human PBL, compared to the other two fractions (Figure 1). 

### 3.2. Quantification of Apoptosis Using Phase-Contrast Microscopy and Propidium Iodide and Acridine Orange Double-Staining

**Figure 2:**
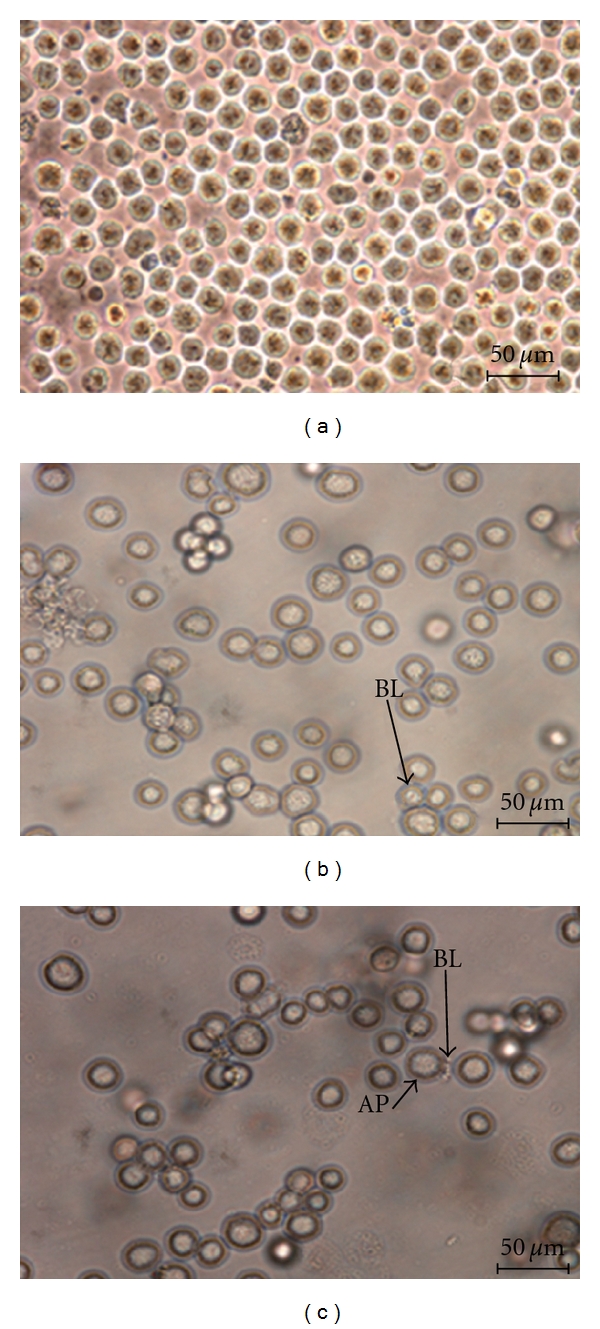
Normal contrast micrographs of CEMss treated at IC_50_ of DCM/F7 in a time-dependent manner. Concentration for 24 hrs (b) and 48 hrs (c) compared with untreated cancer cell (a) (400x magnification). AP: apoptotic cell; BL: blebbing of the cell membrane.

Normal inverted microscopy was carried out to observe any morphological changes occuring in the cells treated with DCM/F7, which was well compared with untreated cells. Figures 2(a)–2(c) showed several morphological changes of treated and untreated cells at 72 h post treatment under ×400 magnification. The treated cells showed obvious changes as compared to untreated cells. Treated CEMss cells showed blebbing of the cell membrane, a more prominent growth inhibition and shrinkage of the cells. On the contrary, untreated cells remained confluent throughout the incubation period.

**Figure 3:**
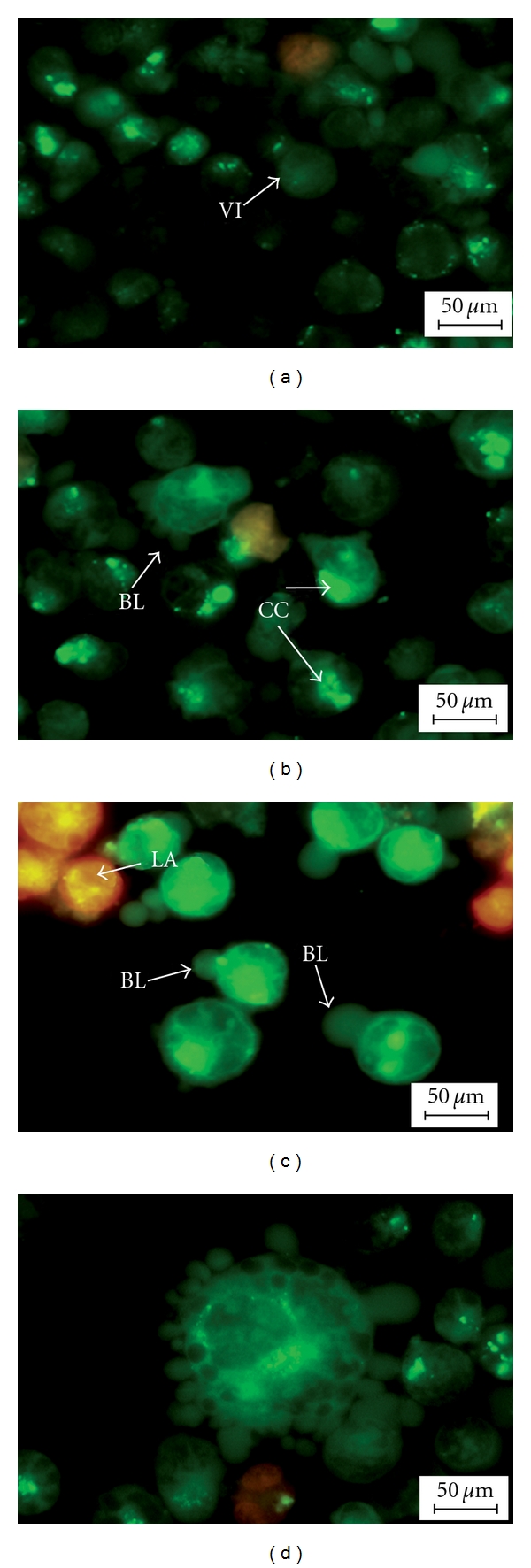
Fluorescent micrographs of acridine orange and propidium iodide double-stained human T4 lymphoblastoid cells (CEMss). CEMss were treated at IC_50_ of DCM/F7 in a time-dependent manner. Cells were cultured in RPMI 1640 media maintained at 37°C and 5% CO_2_. (a) Untreated cells after 48 hr showed normal structure without prominent apoptosis and necrosis. (b) Early apoptosis features were seen after 24 hours representing intercalated acridine orange (bright green) amongst the fragmented DNA, (c, d) Blebbing and orange color representing the hallmark of late apoptosis were noticed in 48-h treatment (magnification 400x). VI: Viable cells; BL: blebbing of the cell membrane; CC: Chromatin condensation; LA: Late apoptosis.

**Figure 4:**
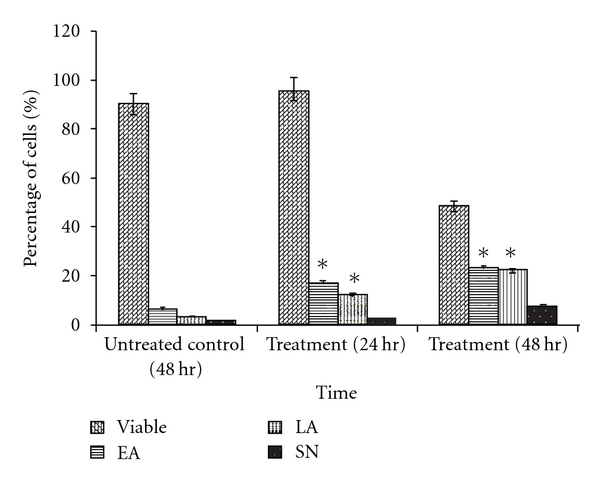
Percentages of viable, early apoptotic, late apoptosis and secondary necrotic cells after DCM/F7 treatment. CEMss cell death via apoptosis increased significantly (*P < .05) in a time-dependent manner. However, no significant (*P* > .05) difference was observed in the cell count of necrosis. Cells were cultured in RPMI 1640 (25 ml flask) media maintained at 37°C and 5% CO_2_. EA: Early apoptosis; LA: Late apoptosis; SN: Secondary necrosis.

Moreover, treated CEMss cells were scored under fluorescence microscope in order to quantify viable, early apoptosis, late apoptotic and secondary necrosis. We counted 200 cells arbitrarily and differentially, together with the untreated negative control. The study revealed that DCM/F7 triggered morphological features that relate to apoptosis in a time-dependent manner (Figures 3(a)–3(d)). Early apoptosis was obvious by intercalated AO within the fragmented DNA. In several of such cases, the fluorescent bright-green color could be seen in treated CEMss cells only. In contrast, untreated cells were observed with a green intact nuclear structure. At 24 h treatment with DCM/F7, blebbing and nuclear chromatin condensation were noticed (moderate apoptosis). In addition, in the late stages of apoptosis, changes such as presence of reddish-orange color due to the binding of AO to denatured DNA were observed after 48 h of treatment. Differential scoring of treated CEMss cells (200 cells population) showed that there is a statistically significant (*P* < .05) difference in apoptosis-positive cells, which indicates clearly that a time-dependent apoptogenic effect has occurred. On the other hand, there was no statistically significant (*P* > .05) difference in necrotic counts at different treatment times (Figure 4). 

### 3.3. Ultramorphological Apoptotic Observations Were Seen in DCM/F7-Treated CEMss Cells

**Figure 5:**
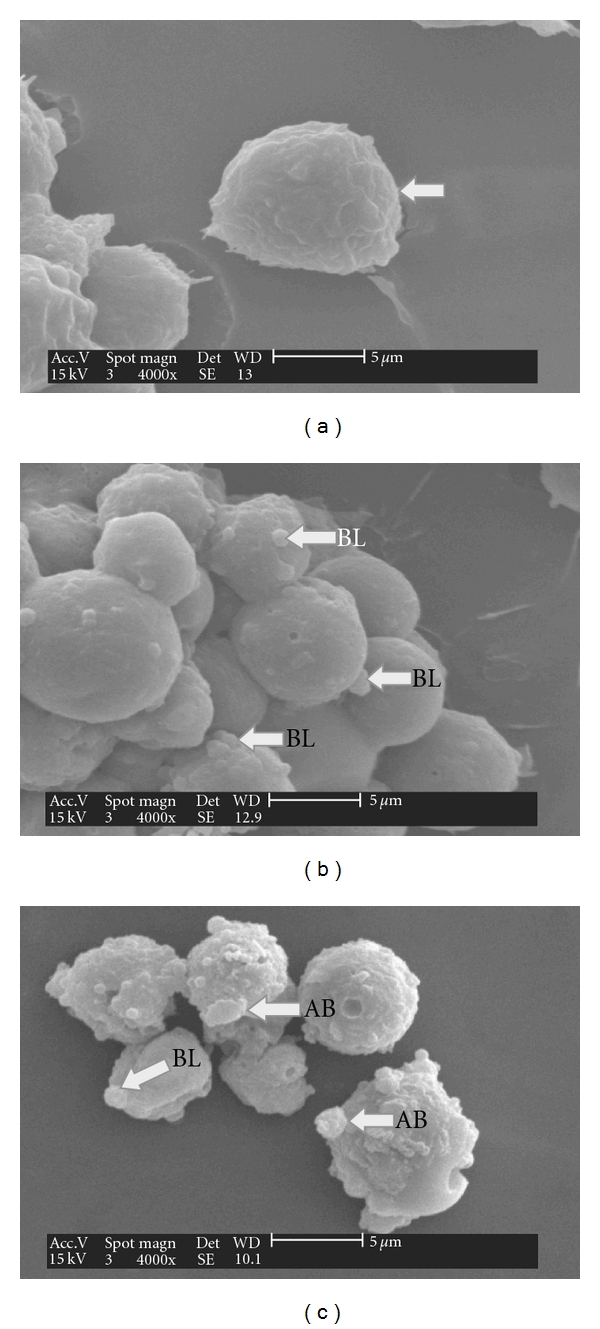
SEM micrographs of surface ultrastructural characteristics of CEMss cells treated with DCM/F7 in a time-dependent manner (24 and 48 h) and cultured in RPMI 1640 media maintained at 37°C and 5% CO_2_. (a) The characteristic of untreated CEMss cells surface showing the restoration of a typical morphological feature of a cancer cell such as smooth surface (as shown in white arrow). (b) and (c) DCM/F7 treated CEMss cells (24 and 48; IC_50_: 3 *μ*g/ml) showed distinct morphological changes corresponding to typical apoptosis, including cell shrinkage and membrane blebbing and separated apoptotic bodies (magnification 4000x). BL: Blebbing; AB: Apoptotic body.

**Figure 6:**
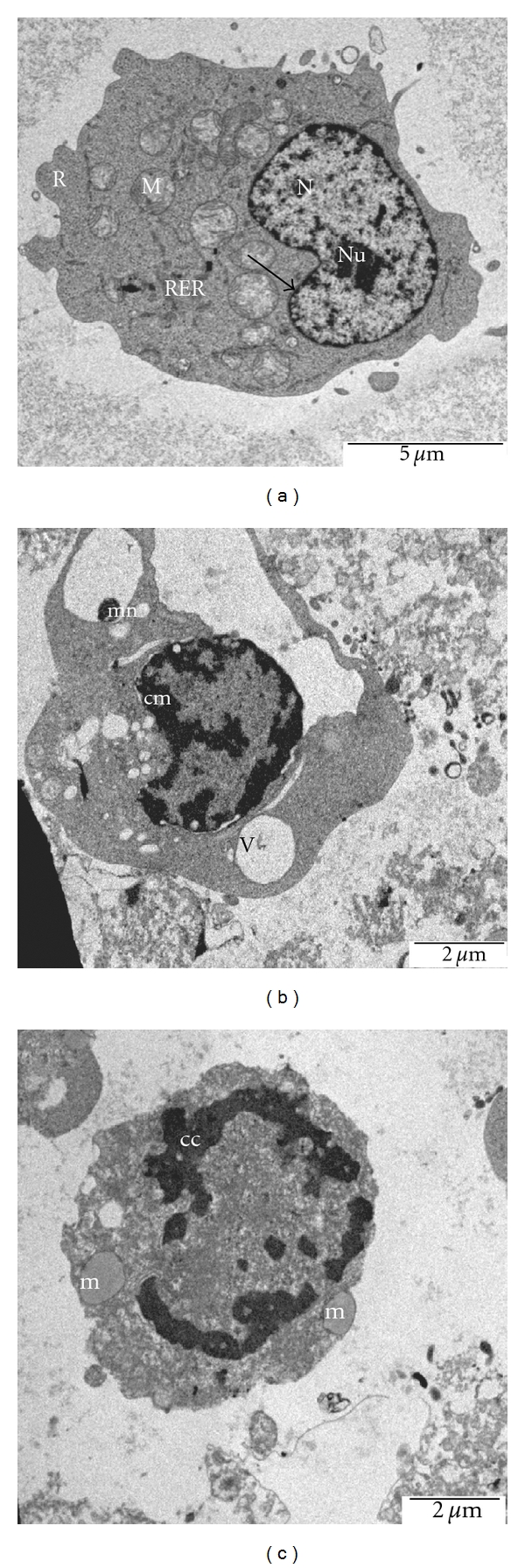
Electromicrograph of untreated human T4 lymphoblastoid leukemia cells (CEMss) demonstrates the normal structure of CEMss cancer cell. The nucleus (N) contains evenly distributed chromatin and a large nucleolus (Nu), mitochondria (M) are large and numerous, rough endoplasmic reticulum (RER), ribosome (R) and the double membrane nuclear envelop (arrow) are also seen (×1000). In the treated cell nuclear chromatin marginisation (**b**-cm) (×1500), chromatin condensation (**c**-cc) (×1500) formation of apoptosis micronuclei (**b**-mn) characterize the apoptosis cell. The vacuolization (v) is also seen. Mitochondria of the control cell (a-m) shows more or less parallel cristae and of a treated cell (c-m) show vacuolization and loss of cristae. Cells were cultured in RPMI 1640 (25 ml flask) media maintained at 37°C and 5% CO_2_.

Morphological analysis of treated CEMss cells was carried out to gain insight into morphological alteration caused by DCM/F7. Both extra- and intracellular structure analysis was carried out by SEM and TEM, respectively. Interpretation of SEM electromicrographs showed distinctive morphological changes corresponding to a typical cellular surface morphology of apoptosis, including cell shrinkage, membrane blebbing and formation of apoptotic bodies (Figures 5(b) and 5(c)). The intramorphological features observed by TEM demonstrated a clear morphological change in the nucleus with the formation of sharply, uniformly and finely granular masses, which marginated against the nuclear envelope (Figures 6(b) and 6(c)). The presence of partly degraded apoptotic bodies around the cells cytoplasm was also evident. All these morphological and ultrastructural changes attributed to the incubation of CEMss cells with 3 *μ*g ml^−1^ DCM/F7 for 24 and 48 h are characteristics associated with apoptosis. On the contrary, untreated CEMss cells showed well-preserved morphology and cell organelles Figures 5(a) and 6(a). These apoptotic effects were found to be time correlated phenomena and this was noticed when considering the number of blebs formation (cytoplasmic extension) as an indicator of cell death via apoptosis. 

### 3.4. ApoBrdU-TUNEL Assay

**Figure 7:**
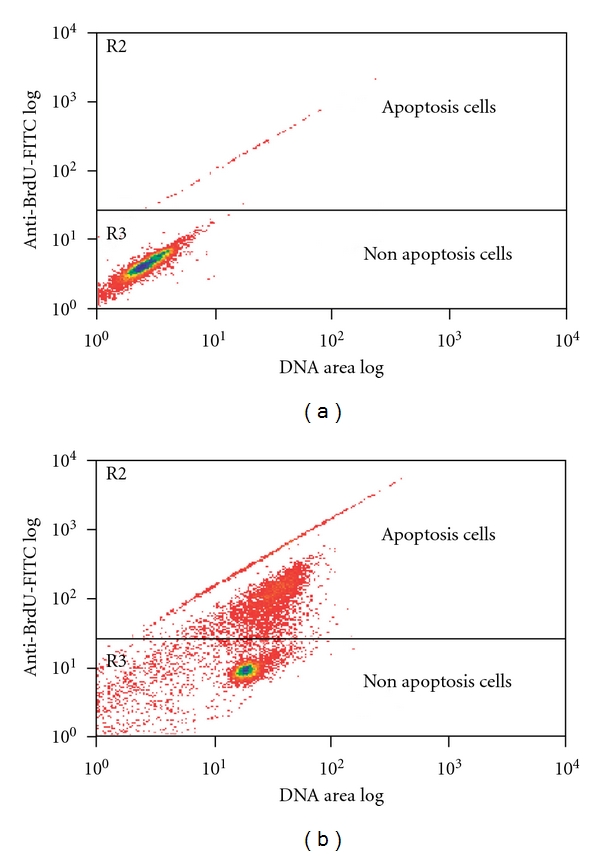
Apo Brdu-TUNEL assay showing the effect of 48 h exposure of CEMss to 3 *μ*g/ml DCM/F7. The *x*-axis of the density plots shows relative DNA content, while *y*-axis shows the relative number of strand break within individual cells. (a) Control 48 h (*n* = 2) and (a) CEMss cells exposed to 3 *μ*g/ml DCM/F7 (*n* = 2 for each experiments).

The biochemical hallmark of apoptosis is the internucleosomal degradation of cellular DNA by an endonuclease, and this results in the appearance of DNA fragments. In order to detect free 3′-OH ends in the DNA fragments, the TUNEL assay method was used and thus apoptotic cells can be identified. The percentages of apoptotic cells determined after DCM/F7 treatments are shown in Figure 7. The significant increase in the number of apoptotic cells with fragmented DNA was observed after the treatment when DCM/F7 was used at the concentration of 3 *μ*g ml^−1^ for 12, 24 and 48 h. In addition, significant time-dependent increase of fragmented DNA was observed at each time point. Our results demonstrated that the number of apoptotic cells increases after 12 h post treatment (Table 2).

### 3.5. Identification and Chemical Analysis of Bioactive Fraction Using GC-MS

Table 3 shows the analysis of the DCM/F7 by GC-MS, which revealed a total of 40 compounds and hesitantly identified 30 compounds including linoleic acid, *n*-hexadecanoic acid, 9 hexadecanoic acid and stigmasta-5,22-dien-3-ol. The unsaturated omega-6 fatty acid, linoleic acid was present in large amounts (51.2%) in DCM/F7 followed by *n*-hexadecanoic acid (17.89%), 9-hexadecanoic acid (6.99%) and stigmasta-5,22-dien-3-ol (6.06%). 

## 4. Discussion

Cancer is the second leading cause of death in the world. The prognosis for a patient with metastatic carcinoma remains a concern and accounts for more than one half of all cancer deaths. Almost all artificial agents currently being used in cancer therapy are known to be toxic and they cause severe damage to normal cells. Therefore, chemoprevention or chemotherapy via nontoxic agents and food supplements could be one approach for decreasing the incidence of these cancers [21]. Recently, CAM has directed its interest toward remedy for vital ailment worldwide [22], which includes herbal medicine [23]. Naturally occurring compounds found in food and medicinal plants could, in theory, serve as alternatives to chemically designed anticancer agents [24] and this kind of drug discovery is relevant to CAM [25].

The effect of *T. flagelliforme* has been studied on human cancer cell lines and in murine P388 [4, 5, 7]. Our results show that this plant has potent cytotoxicity and anticancer potential through inducing apoptosis in CEMss cells. The DCM/F7 showed a concentration-dependent inhibition of cell growth of the CEMss cell line, which was established by MTT assay. The MTT is a standard colorimetric assay (an assay which measures changes in color) for measuring cellular growth. The American National Cancer Institute guidelines set the limit of activity for crude extracts at a 50% inhibition (IC_50_) of proliferation of <30 *μ*g ml^−1^ after an exposure time of 72 h [26]. Therefore, the DCM fractions as well the maximum concentration used in this study were <30 *μ*g ml^−1^. The MTT results showed that fractions DCM/F7, DCM/F11 and DCM/F12 exhibited exceptional activity with 3, 5 and 6.2 *μ*g ml^−1^, respectively, which was below the cytotoxicity level of crude DCM extract. It is critical for an anticancer agent to exhibit high cytotoxicity but such activity should be specific for the cancer cells only. The results obtained from this current investigation indicated that CEMss cells were far more sensitive to the cytotoxic action of DCM/F7 compared to the PBLs. These results were consistent with previous studies, which reported that the fraction obtained from the DCM extract of *T. flagelliforme* showed cytotoxic activity toward NCI-H23 cells, but not to noncancerous BALB/c 3T3 cells [4].

The morphological observation revealed that both extra- and intracellular structures were intensely affected following DCM/F7 treatment. Until today, the microscopic examination has been the gold standard for the most precise detection of apoptosis [27]. With this technique the whole process of apoptosis can be observed and evaluated based on the original morphological criteria [28]. Apoptosis was originally defined by structural alterations in cells observable by various microscopic techniques [29]. The reduction of number of viable cells in the treatment was in accordance with its cytotoxicity property showed the plant fraction. Clear signs of apoptosis, such as cytoplasmic shrinkage and membrane blebbing, were observed by phase contrast microscopy. Moreover, using the properties of AO and PI, CEMss cells were treated with DCM/F7 to determine its antiproliferative and apoptogenic characters. The antiproliferative properties of DCM/F7 could be then evaluated by counting the number of viable cancer cells, while the apoptogenic were determined through observing typical morphological changes of apoptosis. In the present study the cell-viability results showed an obvious decrease of living cells in DCM/F7 treated group. These cells had typical morphological changes associated with apoptosis such as chromatin condensation, DNA fragmentation, membrane blebbing and apoptotic body formation as observed under fluorescence microscopy on AO/PI staining. It is well known that, in apoptosis, the earliest recognized morphological changes are compaction and segregation of the nuclear chromatin, with the result of chromatin margination and condensation of the cytoplasm [30]. Progression of the condensation is accompanied by convolution of the nuclear and cell outlines followed by breaking up of the nucleus into discrete fragments and by budding of the cell as a whole to produce membrane-bounded apoptotic bodies [31]. The cellular events in apoptosis are accomplished very fast, with only a few minutes elapsing between the onset of the process and the cluster formation of apoptotic bodies. In this study, SEM was employed to obtain detailed information about the cell surface of CEMss and ultrastructural changes were studied using TEM. The SEM electron micrograph of current investigation showed distinct morphological changes corresponding to a typical cellular surface morphology of apoptosis, including cell shrinkage, membrane blebbing and formation of apoptotic bodies after treatment. In addition, the TEM observation of treated cell nuclear chromatin marginization, chromatin condensation and formation of vacuoles characterize the apoptosis cell. Besides this, treated cells (CEMss) demonstrate the condensed cristae of mitochondria as a typical morphological feature in apoptosis. In contrast, the untreated cell's nucleus contains evenly distributed chromatin and a large nucleolus, mitochondria are large and numerous, rough endoplasmic reticulum, ribosome and the double membrane nuclear envelops are also seen.

Apoptosis is an active physiological process resulting in cellular self-destruction that involves specific morphological and biochemical changes in the nucleus and cytoplasm. Apoptosis is regulated by two major pathways: the extrinsic (receptor mediated) and the intrinsic (mitochondrial mediated) pathways. Due to the sensitivity of the intrinsic pathway, tumors arise more often through this pathway than the extrinsic pathway [32]. In both ways apoptotic endonucleases degrade chromosomal DNA during programmed cell death. The cleavage of nuclear DNA into nucleosome-sized fragments is a signature of apoptosis. Because such cleavage degrades the genetic materials that are crucial for producing cellular proteins, DNA fragmentation arguably represents the most severe damage to the cell [33]. In the present study the TUNEL assay revealed apoptotic induction in CEMss cells exposed to DCM/F7 3 *μ*g ml^−1^ (IC_50_) at different time point. Similar kind of observations were noted previously when a fraction from DCM extract of this plant induces apoptosis by colorimetric TUNEL assay [4].

The presence of high concentrations of unsaturated aliphatic compounds was clearly evident with most of the identified phytochemicals. This linoleic acid-enriched fraction focusing in to the importance of health supplements which could be act as a drug or prevent the occurrence of malignancies. It is known that high concentration of certain fatty acids can cause cell death via apoptosis or necrosis [34]. Linoleic acid [35–38] as well as conjugated linoleic acid was well documented for its apoptotic effect [39–42]. In addition, Yoo et al. [43] isolated three fatty acids including hexadecanoic acid which has been proven to be a promising anticancer agent to exert apoptosis mediated by caspase-3 activation. Furthermore, the involvement of hexadecanoic acid has been evidenced in the mitochondrial cyclosporin A-insensitive pore induced by Pal/Ca^2+^ complexes in the apoptotic process [44] and Fas-mediated apoptosis in PC12 cells [45]. The rich presence of linoleic acid and its specificity towards cancer cells is in well agreement with the previous studies which demonstrated that dietary conjugated linoleic acid could induce apoptosis of p53 wild-type pre-neoplastic lesions in the rat mammary gland, but not of the normal mammary epithelium [46]. Based on these literatures it can be proposed that the presence of both linoleic acid and hexadecanoic acid might be responsible for the apoptotic effect produced by DCM/F7 on CEMss.

**Figure 8:**
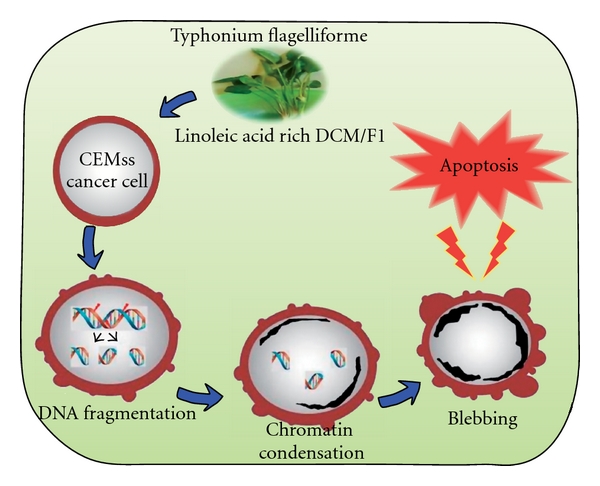
The hypothetical diagram demonstrating the possible apoptotic effect of the DCM/F7 of *T. flagelliforme* in leukemia *in vitro*.

Natural agents that restrain the proliferation of malignant cells by inducing apoptosis may represent a useful mechanistic approach to both cancer chemoprevention and chemotherapy. Thus, there is growing attention in the use of plant materials for the treatment of various cancers and the development of safer and more effective therapeutic agents [47]. Due to additive or synergistic effects, and competing or confounding effects of plant extracts [48], comparison of these effects on well-characterized tumor cell lines provide an outline and model for the discussion and analysis of possible biological mechanisms that produce clinical effects [49, 50]. Similar kind of studies has aided to identify chemotherapeutic agents that provide selective effects against cancerous cells without untoward effects on normal cells [51]. Our study demonstrates that the DCM/F7 fraction of *T. flagelliforme* inhibited leukemia cancer-cell proliferation selectively, via the induction of apoptosis. Apoptosis is a highly regulated and organized cell death process controlling the development and homeostasis of multicellular organisms that occur under a variety of physiological and pathological conditions, which is characterized by a number of well-defined features including cellular morphological change, chromatin condensation and oligonucleosomal DNA cleavage [52]. Our results were shown to be due to apoptosis by the demonstration of the morphological characteristics of apoptosis and the observation of cells containing fragmented nuclei and DNA (Figure 8). These results indicate clearly that *T. flagelliforme* which has been used as a healthcare supplement as well as traditional medicine acts via programmed cell death. In addition, our results emphasize important health benefits of health supplements and herbs containing fatty acids. Additional studies are needed to determine the molecular mechanisms and its pathways of the selected fraction along with its active components and to evaluate this potential anticancer activity *in vivo*. 

## Funding

This research was funded in part by the National Cancer Council (MAKNA), Malaysia. The authors also acknowledge additional support from University Putra Malaysia (UPM), Serdang, Malaysia (Grant no RUGS 91143).

## Figures and Tables

**Table 1:** The yield and growth-inhibitory activity of fractions obtained from DCM extracts of *T. flagelliforme*.

Fractions	Percentage yield value (w/w)	IC_50_ (*μ*g ml^−1^, mean ± SEM)
DCM/F1	5.45	>25
DCM/F2	1.95	>25
DCM/F3	4.6	22 ± 1.02
DCM/F4	3.05	9 ± 0.44
DCM/F5	2.3	16 ± 0.9
DCM/F6	9.85	>25
DCM/F7	10.15	3 ± 0.08
DCM/F8	4.25	21 ± 1.3
DCM/F9	4.98	>25
DCM/F10	4.89	>25
DCM/F11	7.4	5 ± 0.32
DCM/F12	9.65	6.2 ± 0.61

The IC_50_ are average values of three independent triplicate assays.

**Table 2:** Apo Brdu-TUNEL assay of CEMss cells.

	Percentage of cells (%)			
	Control	Treatment		
		12 h	24 h	48 h
Non-apoptotic	98.47 ± 6.2	94.85 ± 6.3	78.96 ± 5.5	71.29 ± 5.9
Apoptotic	1.77 ± 0.2	6.53 ± 0.5	23.05 ± 1.9	29.3 ± 2.1

Apo Brdu-TUNEL assay showing the effect of 48 h exposure of CEMss to 3 *μ*g ml^−1^ DCM/F7. The table shows the effect of control, 12, 24 and 48 h post-treatment TUNEL positive and TUNEL negative cells. ±SD are from two independent experiments.

**Table 3:** Chemical composition of DCM/F7

Pick no	Relative retention time (min)	Component^a^	Relative percentage^b^ (%)
1	9.548	2-Heptinal	0.43 ± 0.03
2	11.729	2-octenal	0.14 ± 0.01
3	12.342	Decane	0.12 ± 0.01
4	14.294	Decane Derivative	0.12 ± 0.02
5	15.665	2-Decenal	0.14 ± 0.01
6	16.282	2,4-Decadienal derivative	0.11 ± 0.01
7	16.73	2,4-Decadienal	0.19 ± 0.02
8	18.245	Vanillin	0.09 ± 0.01
9	20.723	Undecanoic acid	0.49 ± 003
10	22.842	Hexadecanal	0.06 ± 0.01
11	23.032	Tetradecanoic acid derivative	0.15 ± 0.02
12	23.625	Tetradecanoic acid	0.6 ± 0.04
13	23.789	Pentadecanoic acid, methyl ester	0.06 ± 0.01
14	24.254	Hexadecanal	0.09 ± 0.01
15	24.608	Pentadecanoic acid derivative	2.44 ± 0.1
16	24.871	9-Hexadecen-1-ol	0.31 ± 0.03
17	25.013	Pentadecanoic acid	0.54 ± 0.04
18	25.128	1-Tetradecanol	0.42 ± 0.03
19	25.377	9-Hexadecanoic acid	0.12 ± 0.01
20	25.642	Hexadecanoic acid, methyl ester	0.41 ± 0.02
21	26.262	9-Hexadecanoic acid derivative	6.99 ± 0.08
22	26.785	*n*-Hexadecanoic acid	17.89 ± 0.1
23	27.041	*cis*-2-Methyl-7-octadecene	1.99 ± 0.09
24	27.398	Oleic acid	1.49 ± 0.08
25	27.608	Heptadecanoic acid	0.41 ± 0.01
26	27.804	9,12-Octadecadienoic acid, methyl ester	0.79 ± 0.01
27	27.877	11,14,17-Eicosatrienoic acid, methyl ester	0.56 ± 0.01
28	29.199	9,12-Octadecadienoic acid	51.2 ± 0.52
29	44.199	5-Cholestene-3-ol, 24-methyl	1.94 ± 0.08
30	45.232	Stigmasta-5,22-dien-3-ol	6.06 ± 0.07

^a^Components were identified by the comparison of each retention time with authentic standards or diagnosed by the mass spectral databases from NIST/EPA/NIH mass special database library of the GC-MS system, at least with a library fit factor >90%.

^b^Area percentage of individual profiles was used as the relative content of the corresponding components. Values are mean ± SD of triplicate determinations.
